# The role of music therapy in reducing post meal related anxiety for patients with anorexia nervosa

**DOI:** 10.1186/s40337-015-0088-5

**Published:** 2015-12-30

**Authors:** Jennifer Bibb, David Castle, Richard Newton

**Affiliations:** Mental Health CSU, Austin Health, 145 Studley Road, Heidelberg, 3084 Victoria Australia; National Music Therapy Research Unit, Melbourne Conservatorium of Music, University of Melbourne, 151 Barry Street, Parkville, 3010 Victoria Australia; The Body Image & Eating Disorders Treatment & Recovery Service (BETRS), St Vincent’s Hospital, 104 Studley Park Rd, Kew, 3101 Victoria Australia; Department of Psychiatry, University of Melbourne, Parkville, 3010 Victoria Australia

**Keywords:** Music therapy, Anorexia nervosa, Meal support therapy

## Abstract

**Background:**

It is well known that mealtime is anxiety provoking for patients with Anorexia Nervosa. However, there is little research into effective interventions for reducing meal related anxiety in an inpatient setting.

**Methods:**

This study compared the levels of distress and anxiety of patients with Anorexia Nervosa pre and post music therapy, in comparison to standard post meal support therapy. Data was collected using the Subjective Units of Distress (SUDS) scale which was administered pre and post each condition.

**Results:**

A total of 89 intervention and 84 control sessions were recorded. Results from an unpaired t-test analysis indicated statistically significant differences between the music therapy and supported meal conditions.

**Conclusions:**

Results indicated that participation in music therapy significantly decreases post meal related anxiety and distress in comparison to standard post meal support therapy. This research provides support for the use of music therapy in this setting as an effective clinical intervention in reducing meal related anxiety.

## Background

It is well documented that mealtimes are anxiety provoking for patients with Anorexia Nervosa (AN) [1]. Eating and weight gain is associated with feelings of fear and anxiety amongst AN patients [2, 3]. Psychological and physical discomfort are often experienced after meals when patients can become preoccupied with thoughts of purging or feelings of guilt [4]. Support from staff or family during meal times is acknowledged as an important therapeutic activity for decreasing meal-related distress [5–8]. Supported mealtimes are typically facilitated by inpatient staff but there is little research into effective interventions for reducing meal related anxiety in a ward setting [5].

### Music therapy

Participation in music therapy can improve the quality of life, interpersonal relationships and social skills of people with mental illness [9–11]. Music therapy can help promote self-determination and collaboration with patients through focusing on strengths and resource-oriented practice [12]. Literature supports the relationship between music therapy practice in mental health recovery and emphasis on empowerment and patient led processes [12–14]. Use of music therapy to promote feelings of empowerment and equality are arguably expressly important in inpatient mental health settings that may otherwise provide little opportunity for self-determination [15, 16].

### Music therapy and eating disorders

Music therapy may offer motivation for recovery from eating disorders, distraction from negative thoughts and feelings, a sense of autonomy and creative expression [17–19]. Case studies derived from patient experiences have described feelings of renewed self confidence and empowerment through participation in music therapy [20]. In a qualitative study exploring the perceptions of group singing from eight people with eating disorders, participants reported several emotional and cognitive benefits including mental engagement and opportunity to distance themselves from life’s problems [21]. Despite these reports of positive experiences during music therapy, there is no published research examining the role of music therapy during supported meal times. This study aimed to address this gap by evaluating post-meal music therapy amongst a group of inpatients with AN.

## Method

### Setting

The study was conducted in a specialist five bed inpatient eating disorders program situated within an acute psychiatric unit. The program primarily caters for adults with severe anorexia nervosa who have been unable to recover through outpatient treatment. The average age of patients admitted to the inpatient program is 22 years of age, predominately young women. A collaborative conceptualisation-based approach [22] is adopted which is patient-centred and focuses on individualised treatment. The therapy program includes a supported meal time (meal support therapy) that involves a period of post meal distress tolerance and support provided by a team member every lunchtime.

### Design

The aim of this study was to evaluate post-meal music therapy amongst a group of inpatients with AN. The study aimed to both determine if participation in music therapy decreased subjective distress during post-meal support and to understand how participants described their experiences of music therapy during this time. The interest in both understanding and measuring post-meal music therapy suggested a mixed method approach, using different modes of self-reporting on the phenomenon [23]. An embedded mixed methods design was adopted where quantitative and qualitative data were collected within a quasi-experimental design [23]. The qualitative part of the study is not included in this article due to the authors’ desire to conduct a deeper exploration of the participants’ rich descriptions of their experience. The quantitative element which is presented in this article, was a non-randomised pre-post design comparing music therapy with treatment as usual following mealtime. This was considered a fitting design because the intention of the study was to evaluate an existing music therapy program and therefore acted as a pilot study, in an attempt to give indicators of the size of the effect of the clinical intervention and to check the feasibility for a possible larger scale study.

Quantitative data was collected using the Subjective Units of Distress (SUDS) scale which was administered pre and post each intervention and control condition. Participants attended the music therapy intervention twice per week for the duration of their admission. At all other times during the week participants continued with their usual ward program. This project was approved by Human Research Ethics Committee at Austin Health (HREC/14/Austin/75).

### Participants

Adults admitted to the eating disorders program within the acute psychiatric unit, Mental Health Clinical Service Unit at Austin Health were invited to participate in the study. The primary researcher (Bibb) distributed the plain language statement and consent form to patients upon their admission to hospital. Informed consent to participate in the study was obtained from 18 of a total of 32 patients. During this time 89 intervention and 84 control sessions were recorded.

Inclusion criteria for the study were as follows:

- Current patient within the inpatient eating disorder program
- Between 18 and 65 years of age
- Able to read and write (in English) in order to complete data collection tool

Exclusion criteria were:

- Non-English speaking
- Severe cognitive impairment, language problems or hearing impairment

### Materials

**Fig. 1:**
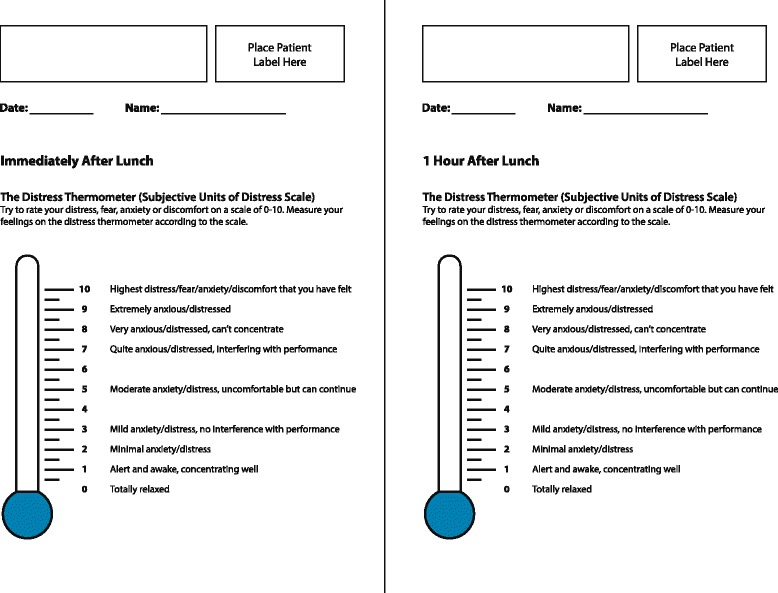
Adapted subjective units of distress thermometers

Participants were trained in the use of the Subjective Units of Distress Scale (SUDS) which is a self-report tool measuring the subjective intensity of distress or anxiety currently experienced by a participant [24]. Although originally used with a 0 to 100 rating scale, more recently scales of 0 to 10 have been adapted, with participants rating their anxiety on a scale ranging from ‘0 – totally relaxed’ to ‘10 – highest distress/anxiety/fear/discomfort you have ever felt.’ A visual analogue scale in the form of a ‘feelings thermometer’ aided in the visual representation of the SUDS ratings (see Fig. 1) [25].

### Procedure

#### Music therapy

Two one-hour music therapy group sessions were held directly after lunch each week. The group was facilitated by a university trained Registered Music Therapist (first author; RMT) in a meeting room on the psychiatric unit. During music therapy sessions participants were encouraged to participate in singing and listening to songs, talking about and sharing music with others and writing songs together. The goal for the group was focused on offering participants a distraction and opportunity to practice coping skills through music. A humanistic approach [26] was adopted where participants were invited to collaborate together on the process of each session. The principles of humanism suggest that “all persons have innate capabilities for actualising their own unique potentials for health and wellbeing” (p.148). Thus, the music therapist maintained a perspective of unconditional positive regard instead of a more directive approach common to cognitive behavioural therapy groups in inpatient eating disorder programs. Group members were encouraged to listen to one another and engage in discussion about song lyrics and their preferred musical tastes. Other topics also emerged in discussions within the group which were often related to eating disorder recovery.

#### Post meal support therapy

Structured post meal support therapy acted as a control condition (treatment as usual) involving a one hour group session after mealtime and occurred on the remaining three days of the working week. These sessions included discussion of feelings, encouragement to focus on achieving the goals of admission and group activities such as playing games or art activities. Nursing and allied health staff facilitated post meal support therapy on a rotating roster.

### Statistical analyses

The statistical software SPSS was used to analyse the data. Mean differences between pre and post scores and the standard deviation were calculated for both intervention and control group data. An unpaired t-test was then performed to explore statistical differences between the mean differences between the music therapy and control interventions.

## Results

A total of 18 patients participated in the study, including 17 females and one male. Participant’s ages ranged between 20 and 58 years old. Length of admission ranged between 21 and 90 days. The 18 participants, attended 173 sessions in total for the music therapy (*n* = 89) and the control conditions (*n* = 84). Results can be seen in Table 1. The mean pre-test score for the intervention group was 8 and the mean post-test score was 5.6. The mean pre-test score for the control group was 8.1 and post-test was 7.1. Mean pre-post test difference for the intervention condition was 2.4 integers on the scale with a standard deviation of 1.9 integers. The mean pre-post test difference for the control condition was 0.93 integers on the scale with a standard deviation of 1.7 integers. The mean standard error for the intervention group was .23 pre-test and .26 post-test, while the mean standard error for the control group was .24 pre-test and .27 post-test. An ANOVA score of f = 28.5 and a highly statistically significant (*p* = <0.0001) difference between the control and intervention conditions was found. The combined mean pre-test score for both control and intervention conditions was higher (8.1) than the mean post-test score for both conditions (6.3), meaning that across all 173 occasions participants rated their anxiety higher prior to participation in either condition, than afterwards. Additionally, there was a statistically significant difference (*p* = <0.0001) between the pre-test scores and post-test scores of all combined 173 occasions.

**Table 1:** Raw data

		Pre-test		Post-test			
Condition	n	Mean	Std error (m)	Mean	Std error (m)	Difference (m)	Std dev.
Intervention	89	8	0.23	5.6	0.26	2.4	1.9
Control	84	8.1	0.24	7.1	0.27	0.93	1.7

## Discussion

The aim of this study was to compare the levels of distress and anxiety of patients with AN pre and post group music therapy provided after meals, with standard post meal support therapy. The results are strongly positive and offer support for the use of music therapy in AN inpatient care. Participants in both conditions reported decreased anxiety post-session compared with straight after lunch (pre-session). This supports previous research suggesting that meal related distress and anxiety is a great concern for patients with AN [1]. Participants’ levels of anxiety significantly decreased after both conditions which also aligns with previous research that suggests therapist facilitated support after meals is helpful for patients with AN [5–8].

Results from the current study also suggest that group music therapy is a more effective intervention for reducing meal related anxiety than standard post meal support therapy in an inpatient setting. The average age of eating disorder patients admitted to the inpatient program is 22 years of age. It is well known that music is an engaging activity for young people and is a motivating factor for participating in therapy [27]. It is likely that participants considered music therapy as a non-threatening and familiar activity in an often confronting medical setting [15, 16]. Participation in music therapy may have acted as a “cognitive divergence” (p. 111) for patients, allowing time for the body to digest food while the mind was attending to something else that was engaging for them [17].

AN is associated with emotion avoidance and dysphoria [28]. Patients report that AN helps them to avoid and control their emotions [29, 30]. Participation in music therapy after meal times is a way for distressing emotions to be experienced through the music. Using music therapy as a distress tolerance technique emphasises the therapy that occurs during the music rather than through discussion [27]. In this instance, the experience of musical process is the therapy [31]. As such, there are broader implications for the use of music therapy as an alternate coping technique for patients who are likely to avoid distressing emotions and often report feeling emotionally ‘numb’ [32].

Participants’ anxiety decreased significantly pre-post the music therapy group compared with standard post meal support therapy. This is important knowledge for inpatient eating disorder programs. Previously it was known that support after mealtime was helpful but the kind of interventions that were effective in reducing anxiety during this time were not [5]. Incorporating music therapy into inpatient meal support programs can offer patients with AN an alternative distress tolerance technique, which they can translate into their external environments post discharge [33]. Previous music therapy research has focused on participants’ experiences of music therapy sessions during their recovery from AN [17–19]. It is important to consider the heightened distress of patients with AN after meal times and the role music therapy can play in reducing anxiety during this time. The current study is the first to use music therapy post meal-time and offers support for further research into this area.

### Limitations

The current study has positive implications for the use of music therapy in reducing meal related anxiety for patients with AN. However, the findings may be limited due to a number of factors. First, the research design was quasi-experimental and did not randomise participants to each condition. The same participants contributed to several occasions (both intervention and control conditions) during their admission which meant randomisation was impossible. Second, participants were recruited from one site which may effect the generalisabiltiy of the results. Third, the same music therapist facilitated the intervention group condition while a variety of therapists (allied health and nursing staff) facilitated the control group condition. The researchers attempted to reduce this potential bias by collecting a large number of occasions (173) over the 36 week period. A larger number of participants, randomisation to control and intervention conditions and recruitment from different hospital sites would benefit future research in this area.

## Conclusions

Music therapy for the treatment of AN is under funded in adult mental health services in Australia. This study provides support for the future funding of inpatient music therapy programs and contributes to the evidence base for the use of music therapy with this population. Music therapy offers an important role in the management of meal related anxiety for patients with AN.
